# Angiopoietin-2 outperforms other endothelial biomarkers associated with severe acute kidney injury in patients with severe sepsis and respiratory failure

**DOI:** 10.1186/s13054-021-03474-z

**Published:** 2021-02-04

**Authors:** Wen-Kuang Yu, J. Brennan McNeil, Nancy E. Wickersham, Ciara M. Shaver, Julie A. Bastarache, Lorraine B. Ware

**Affiliations:** 1 Division of Respiratory Therapy, Department of Chest Medicine, Taipei Veterans General Hospital, Number 201, Section 2, Shipai Road, Beitou District, Taipei City, 11217 Taiwan, ROC; 2 Institute of Physiology, National Yang-Ming University, Taipei, Taiwan; 3 Division of Allergy, Pulmonary and Critical Care Medicine, Department of Medicine, Vanderbilt University Medical Center, T1218 MCN, 1161 21st, Avenue S, Nashville, TN 37232 USA; 4 Department of Cell and Developmental Biology, Vanderbilt University School of Medicine, Nashville, TN USA; 5 Department of Medicine and Department of Pathology, Microbiology and Immunology, Vanderbilt University School of Medicine, T1218 MCN, 1161 21st, Avenue S, Nashville, TN 37232 USA

**Keywords:** Endothelial dysfunction and injury, Sepsis, Angiopoietin-2, Acute kidney injury

## Abstract

**Background:**

Endothelial dysfunction and injury is a major pathophysiologic feature of sepsis. Sepsis is also the most frequent cause of acute kidney injury (AKI) in critically ill patients. Though most studies of AKI in sepsis have focused on tubular epithelial injury, the role of endothelial dysfunction and injury is less well studied. The goal of this study was first to investigate whether endothelial dysfunction and injury biomarkers were associated with severe AKI in sepsis patients. The second goal was to determine the best performing biomarker for severe AKI and whether this biomarker was associated with severe AKI across different etiologies of sepsis and clinical outcomes.

**Methods:**

We studied adults with severe sepsis and acute respiratory failure (ARF) enrolled in the prospective observational Validating Acute Lung Injury markers for Diagnosis (VALID) study. Plasma endothelial dysfunction and injury biomarkers, including angiopoietin-2, soluble vascular endothelial cadherin (sVE-cadherin), endocan and syndecan-1, were measured at study enrollment. Primary analysis focused on the association between endothelial biomarker levels with severe AKI (defined as Kidney Disease: Improving Global Outcomes [KDIGO] AKI stage 2 or 3), other organ dysfunctions (defined by Brussels organ failure scores), and comparison of pulmonary versus non-pulmonary sepsis.

**Results:**

Among 228 sepsis patients enrolled, 141 developed severe AKI. Plasma levels of angiopoietin-2, endocan, sVE-cadherin, and syndecan-1 were significantly higher in sepsis patients with severe AKI compared to those without severe AKI. Among four endothelial biomarkers, only angiopoietin-2 was independently associated with severe AKI (odds ratio 6.07 per log increase, 95% CI 2.34–15.78, *p* < 0.001). Plasma angiopoietin-2 levels by quartile were significantly higher in sepsis patients with hepatic, coagulation, and circulatory failure. Plasma angiopoietin-2 levels were also significantly higher in patients with non-pulmonary sepsis compared to subjects with pulmonary sepsis.

**Conclusion:**

Among four biomarkers of endothelial dysfunction and injury, angiopoietin-2 had the most robust independent association with development of severe AKI in patients with severe sepsis and ARF. Plasma angiopoietin-2 levels were also associated with other organ dysfunctions, non-pulmonary sepsis, and death. These findings highlight the importance of early endothelial dysfunction and injury in the pathogenesis of sepsis-induced AKI.

## Introduction

Sepsis is a syndrome of life-threatening organ dysfunction caused by a dysregulated host response to various infections [1–3]. Although numerous treatments have been studied, sepsis remains one of the most common causes of complications and death among critically ill patients admitted to the intensive care unit (ICU) [4, 5]. The etiology is complex, including both pathogen and host factors, with sites of infection (pulmonary versus non-pulmonary) conferring additional heterogeneity [1, 4]. Endothelial dysfunction and injury plays a fundamental role in the pathogenesis of sepsis, leading to breakdown of the microvascular barrier, loss of homeostasis, and activation of the host innate immune system, culminating in intravascular fluid leakage, interstitial edema, thrombosis formation, vascular dilatation, tissue hypoperfusion, organ dysfunction, and death [6–9].

Acute kidney injury (AKI), an abrupt decrease in renal function as measured by an increase in serum creatinine and/or decrease in urine output, is commonly encountered in the ICU. Sepsis is the most frequent cause of AKI in critically ill patients [10–12], and development of AKI in septic patients results in longer ICU stays, increased costs of hospitalization, and significant long-term morbidity and mortality, especially if the AKI is severe [13–15]. Early detection and aggressive treatment are of vital importance to prevent adverse outcomes. Many studies have focused on the role of acute tubular epithelial injury in sepsis-induced AKI, but the role of endothelial dysfunction and injury is less well understood [16, 17].

Biomarkers of endothelial dysfunction and injury have been extensively studied in the prediction of prognosis and outcomes in critically ill patients with sepsis [18–21]. However, the association between endothelial dysfunction and injury biomarkers and severe AKI in patients with sepsis has not been well studied. The first aim of this study was to explore whether commonly measured endothelial dysfunction and injury biomarkers are associated with severe AKI in patients with sepsis. The second aim was to determine the best performing biomarker for severe AKI and whether this biomarker was independently associated with severe AKI across different etiologies of sepsis and clinical outcomes.

## Methods

### Study design and patient selection

This was a prospective study of patients with age ≥ 18 and severe sepsis as defined by sepsis-2 criteria [22] who were enrolled in the Validating Acute Lung Injury markers for Diagnosis (VALID) study. VALID is a single-center prospective observational cohort study, which has enrolled critically ill patients admitted to four ICUs (surgical, medical, trauma, and cardiovascular) at Vanderbilt University Medical Center (VUMC) since 2006 [23, 24]. Patients were enrolled in the VALID study within 24 h of ICU admission, if there was no plan to discharge from the ICU that day. Clinical data for enrollment day were collected within 24 h prior to enrollment and for the subsequent 72 h for a total of four study days. Patients were excluded from VALID if they had cardiac arrest prior to enrollment, uncomplicated drug overdose, or chronic lung disease requiring home oxygen therapy. After enrollment, plasma was obtained for biomarker measurement on the morning of ICU day 2, within 24 h of ICU admission. Additionally, clinical data and outcomes including patient demographics, Acute Physiology and Chronic Health Evaluation II (APACHE II) scores [25], medical histories, prehospital medications, admission diagnoses, etiology of sepsis (pulmonary vs. non-pulmonary), length of ICU and hospital stay, duration of mechanical ventilation, and hospital mortality were collected comprehensively. Laboratory values, hemodynamic variables, ventilator settings, in-hospital medications, fluid balance, AKI, acute respiratory distress syndrome (ARDS), and evidence of organ failures according to Brussels organ failure scores [26] were recorded for each study day. The study protocol was approved by Vanderbilt Institutional Review Board (IRB #051065). Informed consent was obtained from the patients or surrogates whenever possible; if patients or surrogates were unable to provide consent, the institutional review board granted a waiver of consent due to the minimal risk of the study protocol.

In the current study, to enrich for likelihood of development of severe AKI, patients enrolled in VALID were included if they had severe sepsis according to the sepsis-2 criteria, APACHE II scores equal to or greater than 25, respiratory failure requiring mechanical ventilation, and ICU stay for at least 2 days. For analysis of AKI biomarkers, sepsis patients with known end-stage renal disease requiring regular dialysis were excluded. Patients with pneumonia or aspiration of gastric contents were grouped as pulmonary sepsis, and the remaining patients were grouped as non-pulmonary sepsis. Some patients included in the current study were also included in prior studies of syndecan-1 and soluble vascular endothelial cadherin (sVE-cadherin) levels in severe sepsis [3, 27].

### Definitions of AKI, ARDS, and non-pulmonary organ failure

AKI was determined for each study day using the Kidney Disease: Improving Global Outcomes (KDIGO) serum creatinine criteria during the study period [10]. Briefly, AKI was diagnosed as an increase in serum creatinine ≥ 0.3 mg/dl within 48 h or ≥ 50% times baseline. The baseline creatinine value was determined as the lowest recorded value from the medical record within one year before ICU admission. For patients with no recorded creatinine values in the previous year (*n* = 109), we used the following equation to calculate the assumption of baseline creatinine: [creatinine (in milligrams per deciliter) = 0.74 − 0.2 (if patient is female) + 0.08 (if patient is African ancestry) + 0.003 × age (in years)] [28, 29]. The severity of AKI was determined for each study day by the degree of serum creatinine increase according to the KDIGO AKI criteria. For the current study, KDIGO AKI stage 2 or 3 was categorized as severe AKI. ARDS was assessed daily during the study period using the Berlin definition by two physician investigators who reviewed chest radiographs, blood gases, and clinical data [30]. SpO_2_/FiO_2_ ratio was substituted for PaO_2_/FiO_2_ ratio for the diagnosis of ARDS if blood gas data were not available [23, 31]. Definitions of hepatic, coagulation, and circulatory failure were based on the Brussels organ failure scores [26].

### Measurement of angiopoietin-2, endocan, sVE-cadherin, and syndecan-1

Biomarkers of endothelial dysfunction and injury were measured in duplicate in plasma samples collected on the morning of ICU day 2, within 24 h of ICU admission, using commercially available enzyme-linked immunosorbent assay (ELISA) kits according to the manufacturers’ instructions: angiopoietin-2 (Item No. DY623, R&D Systems, Minneapolis, MN, USA); endocan (Item No. ab213776, Abcam, Cambridge, CB2 0AX, UK); sVE-cadherin (Item No. DCADV0, R&D Systems, Minneapolis, MN, USA); syndecan-1 (Item No. ab46506, Abcam, Cambridge, CB2 0AX, UK).

### Statistical analysis

The primary dependent variable was development of severe AKI within the four study days. Continuous variables were reported as median with interquartile range (IQR) and were analyzed by Mann–Whitney U and Kruskal–Wallis test. Categorical variables were presented as counts and percentages and were analyzed by Chi-square or Fisher’s exact test. Collinearity between these four endothelial biomarkers was analyzed by Spearman rank correlation test. Variables associated with severe AKI, including endothelial dysfunction and injury biomarkers, age, gender, chronic kidney disease (CKD), hepatic failure, coagulation failure, pulmonary sepsis, vasopressor use, and APACHE II scores were included in the univariate analysis. Following univariate analysis, those variables that were found to be associated with severe AKI (*p* < 0.2) in univariate analysis were subjected to multivariate logistic regression analysis using backward elimination to identify the independent risk factors associated with severe AKI. A variable was removed when its removal would cause a change in the exposure odds ratios (ORs) of < 10% at each stage of the backward elimination procedure. Receiver operating characteristic (ROC) curves for plasma levels of angiopoietin-2, sVE-cadherin, endocan, and syndecan-1 to predict the development of severe AKI within the four study days were plotted, and the respective areas under the ROC curves (AUC) were calculated. The association between plasma angiopoietin-2 quartile and categorical outcomes of interest was analyzed by linear-by-linear association test. A *p*-value < 0.05 was considered to be significant. All analyses were performed using SPSS statistics version 19.0 (IBM, Armonk, NY, USA).

## Results

### Patient characteristics and timing and severity of AKI

**Fig. 1:**
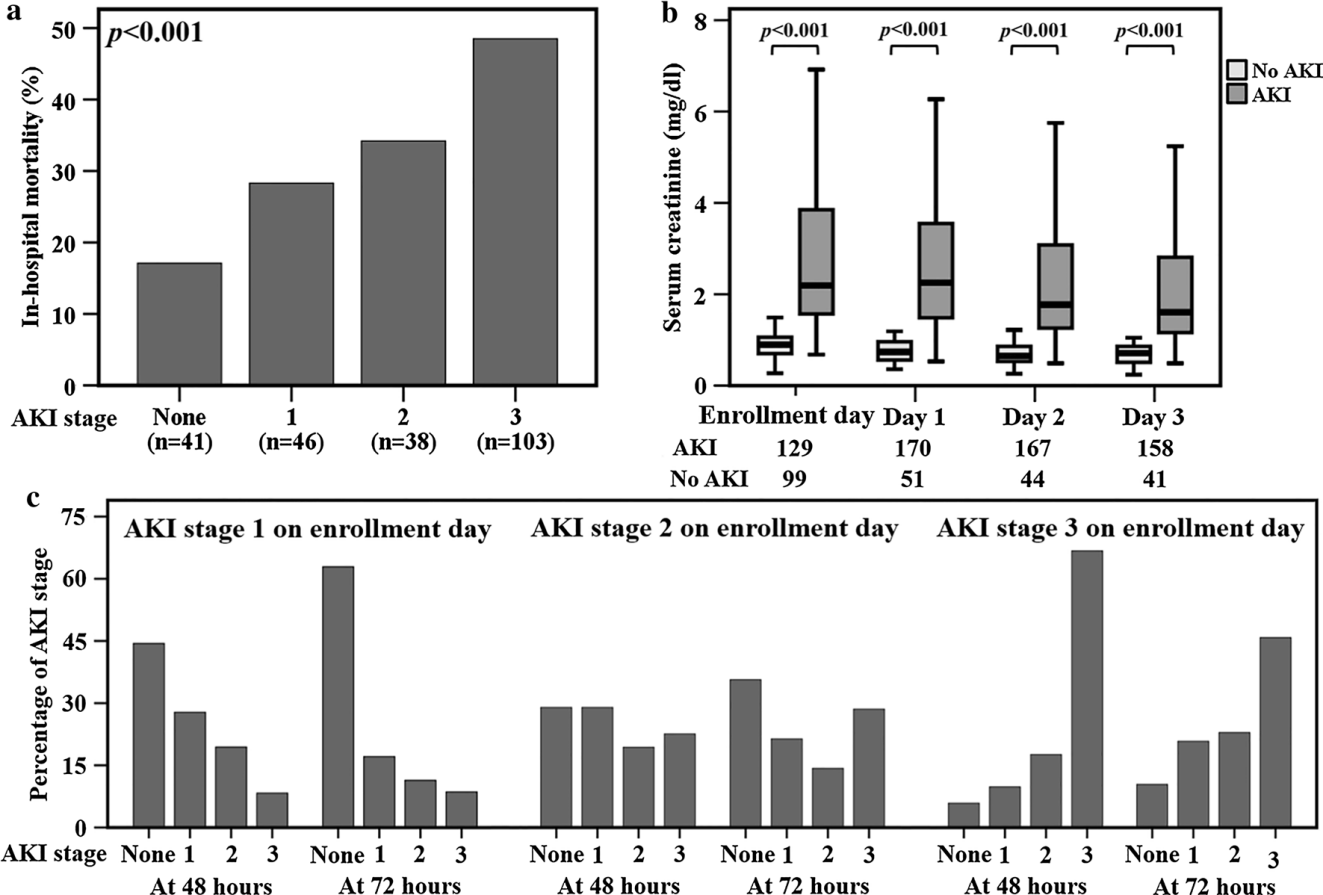
**a** Hospital mortality rates of severe septic patients were significantly associated with severity of AKI. **b** Serum creatinine levels were significantly higher in sepsis with AKI compared to patients without AKI within the four study days. **c** Sepsis patients with AKI stage 1 on enrollment day had a higher proportion of recovery from AKI at 48 or 72 h after enrollment, while a higher percentage of sepsis patients with AKI stage 3 on enrollment day remained in the severe AKI status at 48 or 72 h after enrollment. Groups in panel **a** were analyzed by linear-by linear association test. Data in panel **b** were summarized as boxplots where box encompassed 25‒75th percentile, error bars encompassed 10‒90th percentile and horizontal line showed median. Groups in panel **b** were compared by Mann–Whitney *U* test

There were 228 critically ill patients with severe sepsis and respiratory failure included in this study. The demographic characteristics and clinical parameters are listed in Table 1. In total, 187 (82%) patients had AKI within the four study days. Among patients with AKI within the four study days, 141 (62%) developed severe AKI (KDIGO stage 2 or 3). Patients with severe AKI had a higher APACHE II score (*p* < 0.001), a higher percentage of coagulation failure on enrollment day (*p* = 0.001), pulmonary sepsis (*p* = 0.006), and vasopressor use (*p* = 0.004) compared to sepsis patients without severe AKI. Additionally, patients with severe AKI had significantly fewer ventilator-free days (*p* < 0.001) and a higher hospital mortality rate (*p* = 0.001) compared to severe septic patients who did not develop severe AKI. The hospital mortality rates increased with increasing stage of AKI and were highest in patients with stage 3 AKI (*p* < 0.001, Fig. 1a). The serum creatinine levels within the four study days were significantly higher in sepsis patients with AKI compared to those without AKI (Fig. 1b). Among the 129 patients with AKI on enrollment day, 39 (30%) were stage 1, 38 (29%) were stage 2, and 52 (40%) were stage 3. Patients with AKI stage 1 on enrollment day had a higher proportion of recovery from AKI at 48 or 72 h after enrollment (Fig. 1c). On the other hand, a higher percentage of sepsis patients with AKI stage 3 on enrollment day remained in severe AKI status at 48 or 72 h after enrollment (Fig. 1c).

**Table 1:** Clinical characteristics of 228 critically ill ICU patients with severe sepsis

	Severe AKI (*n* = 141)	No severe AKI (*n* = 87)	*p* value
Age (years)	56 [45–65]	55 [46–66]	0.945
Male	78 (55)	39 (45)	0.135
APACHE II	35 [30–39]	30 [26–32]	< 0.001
Creatinine (mg/dl)	2.03 [1.29–3.74]	0.93 [0.75–1.21]	< 0.001
Ever smoker	87 (62)	53 (61)	1.000
Chronic kidney disease	27 (19)	10 (12)	0.143
Hepatic failure	52 (37)	22 (25)	0.081
Coagulation failure	50 (36)	14 (16)	0.001
Vasopressor use	90 (64)	38 (44)	0.004
ARDS development	69 (49)	42 (48)	1.000
Primary site of infection			
Lung	60 (43)	54 (62)	0.006
Abdomen	18 (13)	5 (6)	0.113
Urogenital tract	17 (12)	6 (7)	0.261
Skin/soft tissue/bone	10 (7)	0 (0)	0.015
Endocarditis/catheter	9 (6)	10 (11)	0.218
CNS/sinus	9 (6)	7 (8)	0.790
Unclear and others	18 (13)	5 (6)	0.113
Ventilator-free days	9 [0–23]	23 [11–26]	< 0.001
ICU days	8 [5–11]	7 [4–11]	0.438
Hospital days	13 [8–22]	11 [8–22]	0.416
Died in hospital	63 (45)	20 (23)	0.001

ICU, intensive care unit; AKI, acute kidney injury; APACHE II, Acute Physiology and Chronic Health Evaluation II; ARDS, acute respiratory distress syndrome

Chronic kidney diseases are based on the medical records

Hepatic failure and coagulation failure are defined by Brussels organ failure scores

Continuous data are expressed as median with interquartile range [IQR], and categorical data are expressed as number of patients (%). *p* value is analyzed by Chi-square test (gender, smoking history, chronic kidney disease, primary site of infection, hepatic failure, coagulation failure, vasopressor use, ARDS development, and mortality) or Mann–Whitney *U* test (age, APACHE II scores, creatinine, ventilator-free days, ICU days, and hospital days)

### Comparison of plasma angiopoietin-2, endocan, sVE-cadherin, and syndecan-1 levels between patients with and without severe AKI

**Fig. 2:**
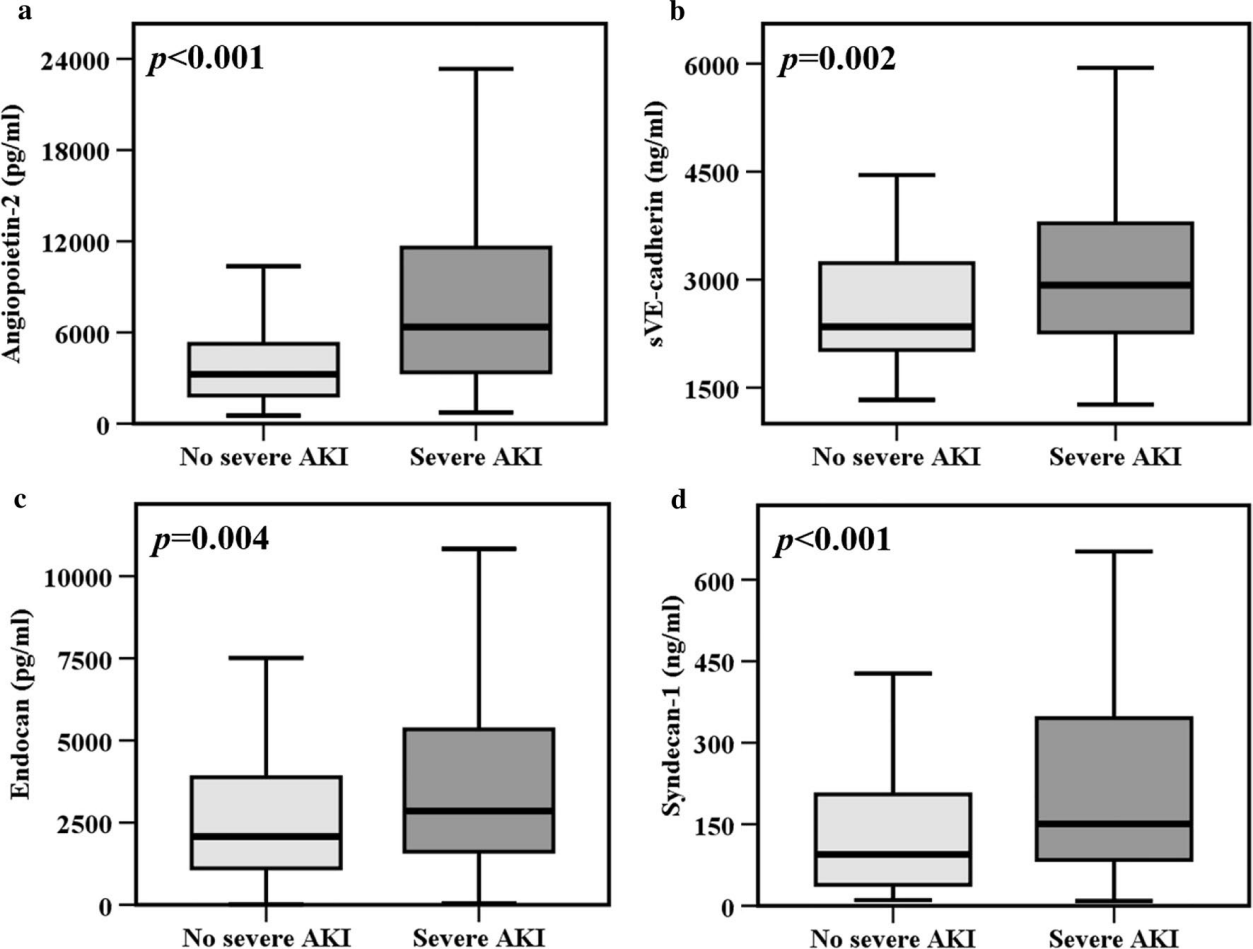
Septic patients with severe AKI had significantly higher plasma levels of **a** angiopoietin-2, **b** sVE-cadherin, **c** endocan and **d** syndecan-1 compared to sepsis patients without severe AKI. Data in panels **a**–**d** were summarized as boxplots where box encompassed 25‒75th percentile, error bars encompassed 10‒90th percentile and horizontal line showed median. Groups in panels **a**–**d** were compared by Mann–Whitney *U* test

Four different biomarkers of endothelial dysfunction and injury were measured and evaluated for their relationship with severe AKI. Patients with severe AKI within the four study days had higher plasma levels of angiopoietin-2 (6372 pg/ml, IQR 3324–12382 pg/ml vs. 3242 pg/ml, IQR 1833–5355 pg/ml, *p* < 0.001, Fig. 2a), sVE-cadherin (2923 ng/ml, IQR 2265–3782 ng/ml vs. 2347 ng/ml, IQR 2013–3337 ng/ml, *p* = 0.002, Fig. 2b), endocan (2859 pg/ml, IQR 1575–5369 pg/ml vs. 2083 pg/ml, IQR 1097–4046 pg/ml, *p* = 0.004, Fig. 2c), and syndecan-1 (150.9 ng/ml, IQR 83.7–347.1 ng/ml vs. 94.7 ng/ml, IQR 38.4–205.8 ng/ml, *p* < 0.001, Fig. 2d) compared to sepsis patients without severe AKI. The ROC curves of the four endothelial dysfunction and injury biomarkers are shown in Additional file 1: Fig. S1. Angiopoietin-2 (AUC = 0.72, 95% CI 0.65–0.79, *p* < 0.001), endocan (AUC = 0.61, 95% CI 0.54–0.69, *p* = 0.004), sVE-cadherin (AUC = 0.62, 95% CI 0.55–0.70, *p* = 0.002), and syndecan-1 (AUC = 0.64, 95% CI 0.57–0.72, *p* < 0.001) all had moderate discriminative powers for severe AKI within the four study days.

### Endothelial dysfunction and injury biomarkers as independent risk factors associated with severe AKI

Collinearity between these four endothelial dysfunction and injury biomarkers is shown in Additional file 2: Fig. S2. Correlations between these endothelial biomarkers were modest. To determine whether endothelial dysfunction and injury biomarkers were independently associated with severe AKI within the four study days, the plasma levels of angiopoietin-2, endocan, sVE-cadherin, and syndecan-1 were log transformed and logistic regression analyses were performed. In univariate analysis, angiopoietin-2 (*p* < 0.001), endocan (*p* = 0.002), sVE-cadherin (*p* = 0.005), and syndecan-1 (*p* = 0.003) were all significantly associated with severe AKI (Table 2). Multivariate logistic regression analysis using backward elimination, including four endothelial dysfunction and injury biomarkers, gender, CKD, hepatic failure, coagulation failure, vasopressor use, pulmonary sepsis, and APACHE II scores, showed that among the four biomarkers, only angiopoietin-2 was an independent risk factor associated with severe AKI within the four study days (odds ratio 6.07 per log increase, 95% CI 2.34–15.78, *p* < 0.001, Table 2).

**Table 2:** Univariate and multivariate logistic regression analyses of risk factors for severe AKI

	Univariate		Simplified model using backward LR elimination method^a^	
	OR (95% CI)	*p* value	AOR (95% CI)	*p* value
Male	1.52 (0.89–2.61)	0.124	_^a^	
Age	1.00 (0.98–1.02)	0.993		
Chronic kidney disease	1.82 (0.84–3.98)	0.132	_^a^	
Vasopressor use	2.28 (1.32–3.93)	0.003	_^a^	
Pulmonary sepsis	0.45 (0.26–0.78)	0.004	_^a^	
Hepatic failure	1.73 (0.96–3.12)	0.071	_^a^	
Coagulation failure	2.87 (1.47–5.59)	0.002	1.99 (0.90–4.42)	0.089
APACHE II	1.26 (1.17–1.35)	< 0.001	1.24 (1.15–1.34)	< 0.001
Angiopoietin-2 (per log increase)	8.81 (3.88–20.01)	< 0.001	6.07 (2.34–15.78)	< 0.001
Endocan (per log increase)	2.29 (1.34–3.91)	0.002	_^a^	
sVE-cadherin (per log increase)	11.87 (2.09–67.43)	0.005	_^a^	
Syndecan-1 (per log increase)	2.73 (1.32–3.93)	0.003	_^a^	

OR, odds ratio; CI, confidence interval; AOR, adjusted odds ratio

^a^Variables that were entered into multivariate logistic regression analysis with backward LR elimination method did not retain the final model

### Plasma angiopoietin-2 levels, development and resolution of AKI

Of 228 severe sepsis patients included, 41 (18%) had no AKI during the study period, 129 (57%) patients had AKI on enrollment day, and 58 (25%) patients developed AKI in the subsequent 72 h (Fig. 1b). The plasma levels of angiopoietin-2 were significantly lower in patients who had no AKI within the four study days (2639 pg/ml, IQR 1422–5408 pg/ml) compared to patients who developed AKI in the subsequent 72 h after enrollment (4255 pg/ml, IQR 2656–7169 pg/ml) or patients who had AKI on enrollment day (5844 pg/ml, IQR 2693–11,447 pg/ml, Additional file 3: Fig. S3a). Among 129 patients with AKI on enrollment day, 90 and 74 patients remained in the AKI status at 48 or 72 h after enrollment, respectively. Omitting patients from the analysis whose AKI resolved by 48 or 72 h, the plasma levels of angiopoietin-2 remained significantly lower in patients without any AKI within the four study days compared to patients with unresolved AKI at 48 (6395 pg/ml, IQR 2706–11,985 pg/ml) or 72 (6395 pg/ml, IQR 3000–11,514 pg/ml) hours after enrollment (Additional file 3: Fig. S3b). Furthermore, the plasma levels of angiopoietin-2 were significantly lower in those who had resolution of AKI in the subsequent 48 or 72 h compared to patients who had persistent AKI at 48 h (4221 pg/ml, IQR 1854–5966 pg/ml vs. 6395 pg/ml, IQR 2706–11,985 pg/ml, *p* = 0.027) or 72 h (3805 pg/ml, IQR 1744–6475 pg/ml vs. 6395 pg/ml, IQR 3000–11,514 pg/ml, *p* = 0.008) after enrollment (Additional file 3: Fig. S3c).

### Plasma angiopoietin-2 levels, severity of AKI and cumulative fluid balance

**Fig. 3:**
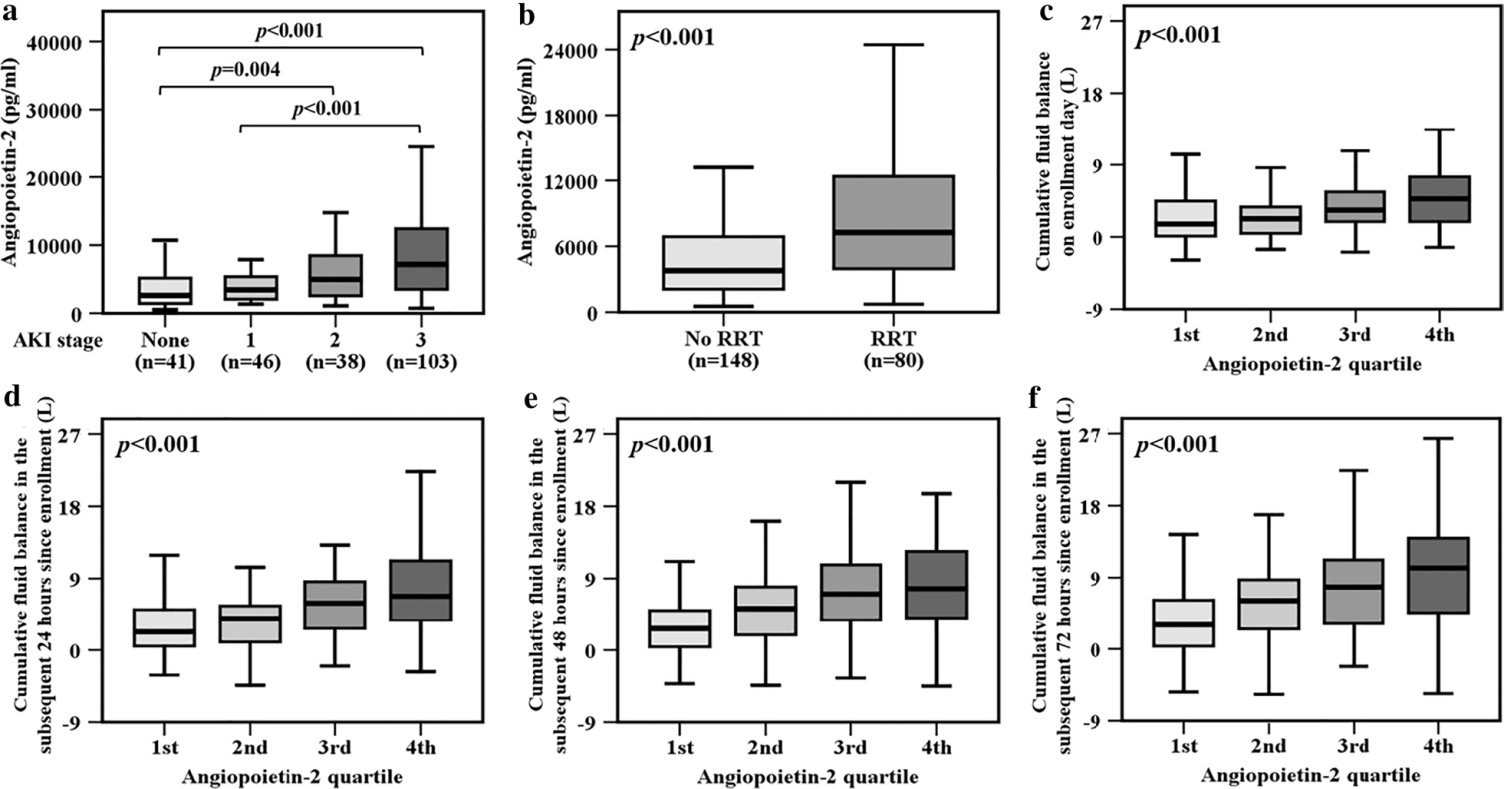
**a** Plasma angiopoietin-2 levels were significantly associated with severity of AKI. **b** Patients requiring renal replacement therapy (RRT) had significantly higher plasma angiopoietin-2 levels than those not requiring RRT during hospitalization. Higher plasma angiopoietin-2 levels by quartile were significantly associated with positive fluid balance **c** on enrollment day, **d** in the subsequent 24 h, **e** 48 h and **f** 72 h since enrollment. Data in panels **a**–**f** were summarized as boxplots where box encompassed 25‒75th percentile, error bars encompassed 10‒90th percentile and horizontal line showed median. Groups were compared by Kruskal–Wallis test (panels **a** and **c**–**f**) or Mann–Whitney *U* test (panel **b**). Post hoc analysis of groups comparison was performed using Mann–Whitney *U* test and Bonferroni correction (panel **a**)

Because angiopoietin-2 had the strongest association with severe AKI of the biomarkers tested in the current study and was associated sepsis-induced endothelial permeability in previous studies [18, 35, 36], we further analyzed the relationship between angiopoietin-2, severity of AKI and cumulative fluid balance in greater depth. Plasma angiopoietin-2 levels increased with stage of AKI and were highest in patients with stage 3 AKI (*p* < 0.001, Fig. 3a). Patients who required renal replacement therapy during hospitalization had significantly higher levels of angiopoietin-2 compared to those who did not (*p* < 0.001, Fig. 3b). Higher plasma angiopoietin-2 levels by quartile at enrollment were significantly associated with a higher positive fluid balance within the four study days (Fig. 3c–f). Moreover, patients with more severe AKI had a higher cumulative fluid balance within the four study days (Additional file 4: Fig. S4).

### Plasma angiopoietin-2 levels, etiology of sepsis and severe AKI

**Fig. 4:**
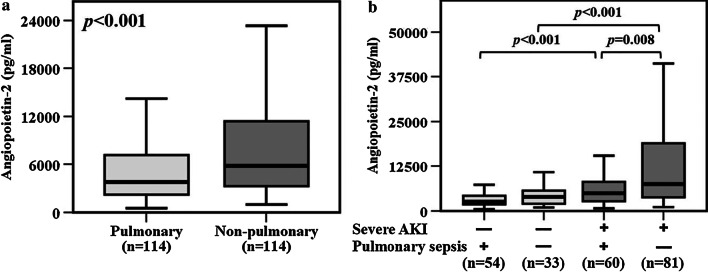
**a** Plasma angiopoietin-2 levels were significantly higher in patients with non-pulmonary sepsis compared to those with pulmonary sepsis. **b** Plasma angiopoietin-2 levels were significantly higher in both pulmonary and non-pulmonary sepsis patients with any severe AKI during the study period compared to those without severe AKI during the study period. Data in panels **a** and **b** were summarized as boxplots where box encompassed 25‒75th percentile, error bars encompassed 10‒90th percentile and horizontal line showed median. Groups were compared by Mann–Whitney *U* test (panel **a**) or Kruskal–Wallis test (panel **b**). Post hoc analysis of groups comparison was performed using Mann–Whitney *U* test and Bonferroni correction (panel **b**)

Previous studies reported that endothelial dysfunction and injury were more profound in non-pulmonary sepsis [2, 3]. In our study, plasma angiopoietin-2 levels were also significantly higher in non-pulmonary sepsis patients compared to those with pulmonary sepsis (*p* < 0.001, Fig. 4a). Next, we sought to determine whether plasma angiopoietin-2 levels were associated with severe AKI in both pulmonary and non-pulmonary sepsis. In patients with pulmonary sepsis, plasma angiopoietin-2 levels were higher in patients with severe AKI compared to those without severe AKI (*p* < 0.001, Fig. 4b). In patients with non-pulmonary sepsis, plasma angiopoietin-2 levels were also higher in patients with severe AKI compared to those without severe AKI (*p* < 0.001, Fig. 4b). Furthermore, plasma angiopoietin-2 levels were higher in severe AKI patients with non-pulmonary sepsis compared to those with pulmonary sepsis (*p* = 0.008, Fig. 4b).

### Plasma angiopoietin-2 levels, other organ failures and mortality

**Fig. 5:**
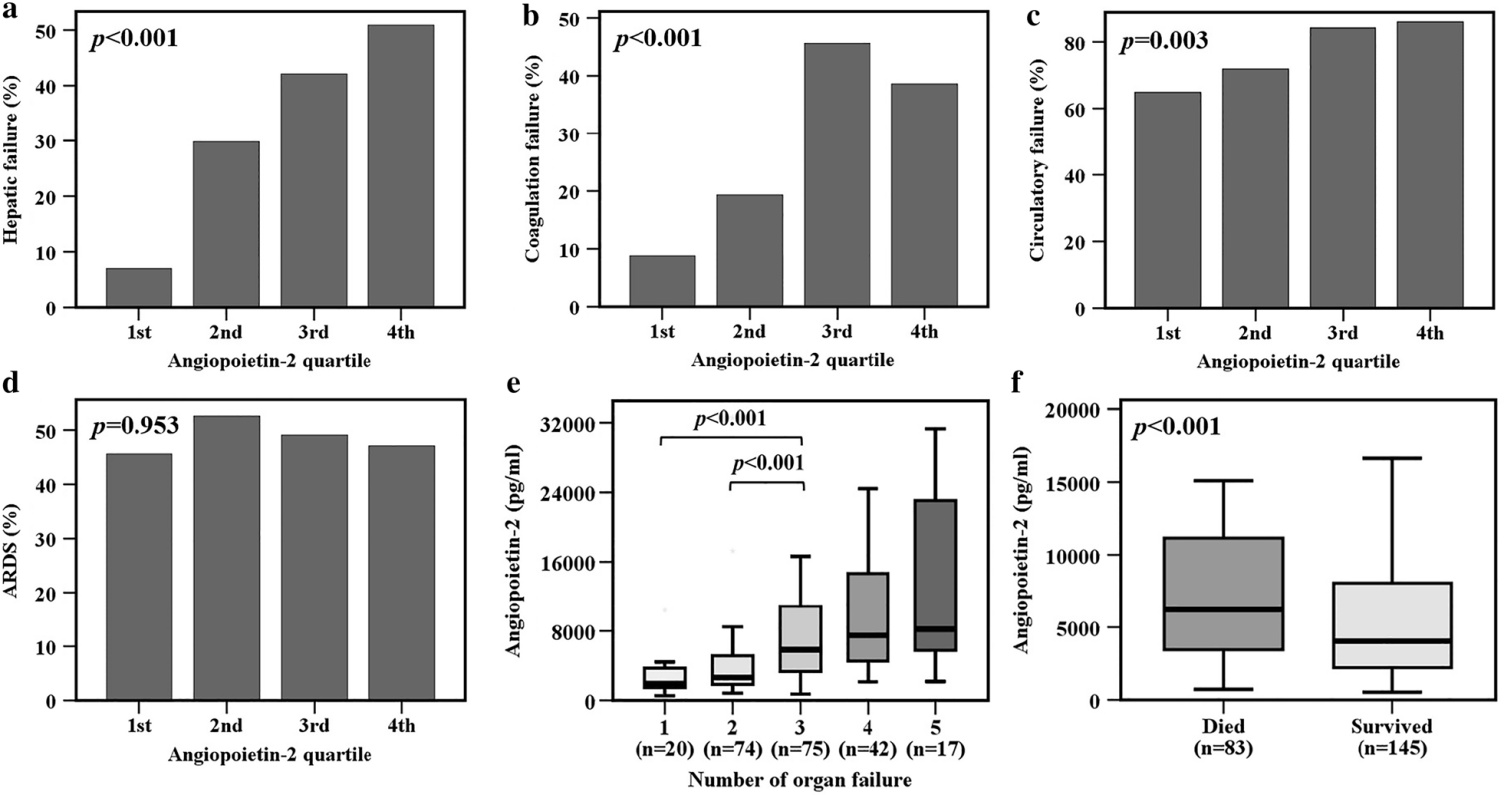
Higher plasma angiopoietin-2 levels by quartile were significantly associated with **a** hepatic failure, **b** coagulation failure and **c** circulatory failure on enrollment day. Organ failures were defined by Brussels organ failure scores. **d** Plasma angiopoietin-2 levels were not associated with the development of ARDS defined by Berlin criteria during the study period. **e** Plasma angiopoietin-2 levels were associated with the number of organ failures on enrollment day. **f** Severe sepsis patients who died during hospitalization had significantly higher plasma angiopoietin-2 levels compared to those who survived. Groups in panels **a**–**d** were analyzed by linear-by linear association test. Data in panels **e** and **f** were summarized as boxplots where box encompassed 25‒75th percentile, error bars encompassed 10‒90th percentile and horizontal line showed median. Groups were compared by Kruskal–Wallis test (panel **e**) or Mann–Whitney *U* test (panel **f**). Post hoc analysis of groups comparison was performed using Mann–Whitney *U* test and Bonferroni correction (panel **e**)

To determine whether plasma angiopoietin-2 levels were also associated with non-kidney organ dysfunction, we compared quartiles of angiopoietin-2 levels to indices of non-kidney organ dysfunction. Higher plasma angiopoietin-2 levels by quartile were significantly associated with hepatic failure (*p* < 0.001, Fig. 5a), coagulation failure (*p* < 0.001, Fig. 5b), and circulatory failure (*p* = 0.003, Fig. 5c) as defined by Brussels organ failure scores. Plasma angiopoietin-2 levels were not associated with the development of ARDS (*p* = 0.953, Fig. 5d). Plasma angiopoietin-2 levels were significantly higher in patients with a greater number of organ failures (*p* < 0.001, Fig. 5e). Moreover, higher plasma angiopoietin-2 levels by quartile were significantly associated with higher APACHE II scores (*p* = 0.008, Additional file 5: Fig. S5). Patients who died in hospital had higher plasma angiopoietin-2 levels compared to sepsis patients who survived (*p* < 0.001, Fig. 5f).

## Discussion

Endothelial dysfunction and injury is a major pathophysiologic hallmark of sepsis, leading to production and release of a variety of biomarkers from the endothelium into the circulation [8, 19]. In the present study, we found that several endothelial dysfunction and injury biomarkers, including angiopoietin-2, endocan, sVE-cadherin, and syndecan-1, were higher in sepsis patients who developed severe AKI. Of these four biomarkers, angiopoietin-2 had the most robust association with severe AKI. Furthermore, plasma angiopoietin-2 levels were significantly higher in both sepsis patients with more organ failures and those who died in the hospital. These findings indicate that sepsis-induced endothelial dysfunction and injury at ICU admission as measured by angiopoietin-2 is associated with an increased risk of adverse outcomes, including AKI, multiple organ failures, and death.

Angiopoietin-2 is produced in endothelial cells and pre-stored in the Weibel-Palade bodies together with von Willebrand factor (vWF). Kümpers et al. reported that protein expression of angiopoietin-2 was prominent in the glomerular endothelium in patients with lupus nephritis compared with healthy subjects and that the serum levels of angiopoietin-2 were closely correlated with the severity of systemic lupus erythematosus [32]. In addition, serum levels of angiopoietin-2 were significantly elevated in ANCA-associated vasculitis with renal involvement compared to patients with active limited granulomatous disease restricted to the respiratory tract or healthy subjects [33]. Taken together, these findings suggest that angiopoietin-2 may be released from the renal endothelium when the kidneys suffer an inflammatory injury. In the setting of sepsis, angiopoietin-2 is released into the circulation where it may have autocrine effects that include disruption of the endothelial barrier and increased microvascular permeability [8, 19, 34]. AKI is one of the most common sepsis-related organ dysfunctions [10–12]. Robinson-Cohen et al. reported plasma levels of endothelial biomarkers, especially angiopoietin-2, were significantly increased in critically ill patients with AKI, independent of inflammation, and angiopoietin-2 remained associated with the development of later onset (24 h later) AKI [35]. Our present study, in concordance with previous reports, demonstrated plasma angiopoietin-2 levels at enrollment were not only significantly higher in severe sepsis patients with AKI, but also were associated with AKI severity including both KDIGO AKI stage and need for renal replacement therapy. Vascular endothelial cadherin (VE-cadherin) is the major proteinaceous component of adherens junctions between endothelial cells [3]. Endocan is a proteoglycan secreted by activated endothelium, and syndecan-1 is a proteoglycan component of the endothelial glycocalyx [27, 36]. The results of the present study also demonstrated that increased shedding of VE-cadherin, endocan and syndecan-1 from the endothelium into the circulation is associated with severe AKI. However, in multivariable analysis, only angiopoietin-2 was independently associated with severe AKI within the four study days, even when controlling for severity of illness and other organ dysfunctions. Release of angiopoietin-2 from dysfunctional endothelial cells into the circulation may precede profound endothelial injury, including disruption of adherens junctions and shedding of endothelial proteoglycan into the circulation. A rise in plasma angiopoietin-2 levels could be a biomarker for early detection of severe AKI, but larger studies are needed.

Ziegler et al. reported that angiopoietin-2 modulated endothelial dysfunction and injury, including microvascular permeability, in a lipopolysaccharide-induced murine sepsis model [37]. Fisher and colleagues found that higher plasma baseline levels of angiopoietin-2 were significantly associated with fluid overload, an index of microvascular permeability and leakage, within the first six hours in patients with development of septic shock [38]. Our current study further showed that plasma levels of angiopoietin-2 were not only strongly correlated with positive fluid balance within the four study days in patients with severe sepsis and respiratory failure, but also inversely associated with the ventilator-free days (*p* < 0.001, Spearman’s rho = -2.33). These findings support the concept that angiopoietin-2 levels may be an indicator of more profound sepsis-induced microvascular permeability that results in more fluid leakage from the circulation into the interstitium, more severe pulmonary edema and requirement for longer mechanical ventilation. Kümpers et al. [39] and David et al. [40] reported circulating angiopoietin-2 levels were associated with multiple organ dysfunction, disease severity and death in sepsis patients. In the current study, we extended these findings to sepsis patients requiring mechanical ventilator support for respiratory failure and found that plasma angiopoietin-2 levels were not only correlated with the disease severity, including the number of organ failures and APACHE II scores, but also were significantly higher in sepsis patients who died in the hospital. Taken together, these findings reveal that the severity of endothelial dysfunction and injury is closely associated with the severity of sepsis and its outcomes.

Sepsis is a heterogeneous clinical syndrome with wide variability in both the type of underlying infection and the primary site of infection. We previously reported that VE-cadherin shedding was more severe in patients with non-pulmonary sepsis compared to those with pulmonary sepsis [3]. Murphy et al. reported that ARDS patients with non-pulmonary sepsis had higher levels of plasma syndecan-1 compared to those with pulmonary sepsis [27]. The present study adds to these prior studies, demonstrating that plasma angiopoietin-2 levels were also higher in non-pulmonary sepsis compared to pulmonary sepsis. These findings suggest that biomarkers of endothelial dysfunction and injury such as angiopoietin-2 could have a role in prognostic and predictive enrichment for future studies of sepsis therapies that focus on ameliorating endothelial dysfunction and injury in patients with non-pulmonary sepsis. The need for new approaches to reduce heterogeneity in clinical trials in sepsis and other critical illness syndrome has been identified as a research priority by the National Heart Lung and Blood Institute [41].

The pathophysiology of sepsis-induced AKI is complex and multifactorial, including inflammation, renal tubular epithelial and glomerular endothelial cellular responses to the inflammatory insult, and microvascular dysfunction [16, 17, 42]. Cross-talk with other organs, especially the lung, has also been reported to predispose to AKI in human and animal studies [43–45]. In the current study, we included only more severely ill patients with respiratory failure requiring mechanical ventilation and high severity of illness as measured by APACHE II score equal to or greater than 25. Given the uniform presence of respiratory failure in all patients included in the current study, the role of lung–kidney cross-talk in development of severe AKI in this study cannot be determined.

Our study has several limitations. First, the VALID study is a single-center, prospective, and observational cohort study, which has enrolled critically ill patients admitted to Vanderbilt Medical, Surgical, Trauma and Cardiovascular ICUs since 2006. Treatment strategies for patients with sepsis and respiratory failure might not be consistent over a 15-year period, including mechanical ventilator settings, antibiotic use, intravenous fluid resuscitation and timing of renal replacement therapy for AKI. A larger multi-center study to measure plasma angiopoietin-2 levels should be performed to validate these results. Second, the VALID study was not designed for AKI as a primary endpoint. Hourly urine output data were not collected, and thus, AKI was only determined by changes in serum creatinine. Furthermore, 109 (48%) patients in the current study required estimation of baseline creatinine because they had no baseline creatinine recorded in the previous year. Therefore, the incidence and severity of AKI might not be fully and exactly captured. Of note, there were no significant differences in the occurrence of severe AKI between patients with known vs estimated baseline creatinine (68% vs. 58%, respectively, *p* = 0.275). In addition, a large majority of patients enrolled in the current study developed AKI within 24 h. Whether these endothelial biomarkers predict later onset of AKI is not clear and will require further study. Third, this study only enrolled severe sepsis patients with APACHE II scores equal to or greater than 25 and respiratory failure requiring mechanical ventilation. For this reason, further studies should be carried out to unravel these controversies in less severely ill septic patients, septic patients without respiratory failure, or critically ill patients without sepsis. Fourth, the mechanisms of plasma angiopoietin-2 clearance remain unclear and have not been well studied. Elevation of plasma angiopoietin-2 levels or other endothelial biomarkers might also be due to impairment of clearance in the setting of organ dysfunction. Fifth, endothelial biomarkers were only measured on the morning of ICU day 2. Whether kinetic changes in these endothelial biomarkers over time are associated with disease improvement or progression is not known. Plasma levels of angiopoietin-2 and other endothelial biomarkers should be measured serially in future studies to clarify these issues. Finally, as this was an observational study, no conclusions about a causal role for angiopoietin-2 in the pathogenesis of sepsis-induced AKI can be made.

## Conclusion

In conclusion, the present study demonstrates that among four plasma biomarkers of endothelial dysfunction and injury, the best performing biomarker independently associated with severe AKI is angiopoietin-2. Plasma angiopoietin-2 levels were also associated with hepatic failure, coagulation failure, circulatory failure and the overall number of organ dysfunctions. Patients who died and patients with non-pulmonary sepsis had higher plasma angiopoietin-2 levels compared to those who survived or who had pulmonary sepsis. Furthermore, sepsis patients with severe AKI had higher plasma angiopoietin-2 levels regardless of whether sepsis was pulmonary or non-pulmonary. Taken together, these findings suggest that endothelial dysfunction and injury as measured by circulating angiopoietin-2 levels is more extensive in sepsis patients with severe AKI.

## Supplementary Information

**Additional file 1.** Receiver operator curves of plasma levels of angiopoietin-2, endocan, sVE-cadherin and syndecan-1 predicted the development of severe AKI within the four study days. AUC, area under the curve.

**Additional file 2.** Plasma levels of angiopoietin-2 were modestly associated with (**a**) plasma levels of endocan, (**b**) plasma levels of syndecan-1 and (**c**) plasma levels of sVE-cadherin. Plasma levels of endocan were modestly associated with (**d**) plasma levels of syndecan-1, but not associated with (**e**) plasma levels of sVE-cadherin. Plasma levels of sVE-cadherin were modestly associated with (**f**) plasma levels of syndecan-1. Collinearity of these four endothelial biomarkers was analyzed by Spearman rank correlation test.

**Additional file 3.** (**a**) Plasma levels of angiopoietin-2 were significantly lower in sepsis patients who did not develop AKI within the four study days compared to patients who had AKI on enrollment day or patients who developed AKI in the subsequent 72 hours after enrollment. (**b**) Plasma levels of angiopoietin-2 were significantly lower in sepsis patients without any AKI within the four study days compared to sepsis patients with persistent AKI at 48 or 72 hours since enrollment. (**c**) Among patients with AKI on enrolment day, plasma levels of angiopoietin-2 were significantly lower in patients with the resolution of AKI compared to patients without the resolution of AKI at 48 hours or 72 hours after enrollment. Data in panels **a**–**c** were summarized as boxplots where box encompassed 25‒75th percentile, error bars encompassed 10‒90th percentile and horizontal line showed median. Groups were compared by Kruskal-Wallis test (panels **a** and **b**) or Mann-Whitney U test (panel c). Post hoc analysis of groups comparison was performed using Mann-Whitney U test and Bonferroni correction (panels **a** and **b**).

**Additional file 4.** (**a**) The severity of AKI within the four study days was not associated with positive cumulative balance on enrollment day. The severity of AKI within the four study days was significantly associated with positive fluid balance in the subsequent (**b**) 24 hours, (**c**) 48 hours and (**d**) 72 hours since enrollment. Data in panels a-d were summarized as boxplots where box encompassed 25‒75th percentile, error bars encompassed 10‒90th percentile and horizontal line showed median. Groups were compared by Kruskal-Wallis test (panels **a**–**d**).

**Additional file 5.** Higher plasma angiopoietin-2 levels by quartile were associated with higher APACHE II scores. Data were summarized as boxplots where box encompassed 25‒75th percentile, error bars encompassed 10‒90th percentile and horizontal line showed median. Groups were compared by Kruskal-Wallis test. Post hoc analysis of groups comparison was performed using Mann-Whitney U test and Bonferroni correction.

## Data Availability

The data used and analyzed during the current study are available from the corresponding author on reasonable request.

## Abbreviations

AKI: Acute kidney injury
VALID: Validating Acute Lung Injury markers for Diagnosis
ARDS: Acute respiratory distress syndrome
ICU: Intensive care unit
CKD: Chronic kidney disease
APACHE II: Acute Physiology and Chronic Health Evaluation II
KDIGO: Kidney Disease: Improving Global Outcomes
IQR: Interquartile range
ELISA: Enzyme-linked immunosorbent assay
VWF: Von Willebrand factor
