# Supplementary figures and images for: Angiopoietin-2 outperforms other endothelial biomarkers associated with severe acute kidney injury in patients with severe sepsis and respiratory failure
Source: Crit Care. 2021 Feb 4;25:48. doi: 10.1186/s13054-021-03474-z (PMC7859898; doi:10.1186/s13054-021-03474-z)

**Supplementary figure 1**

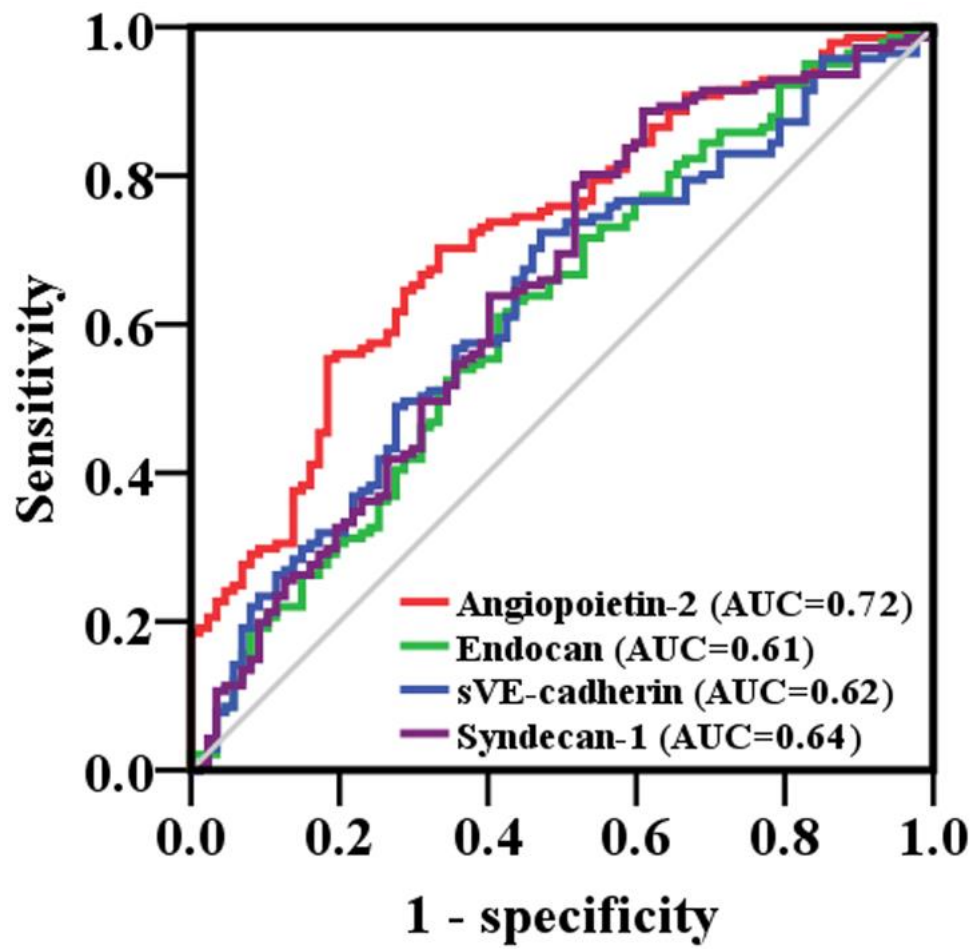

Supplement: Supplementary file 1 — Additional file 1. Receiver operator curves of plasma levels of angiopoietin-2, endocan, sVE-cadherin and syndecan-1 predicted the development of severe AKI within the four study days. AUC, area under the curve. [file 13054_2021_3474_MOESM1_ESM.pdf]

Supplementary figure 2

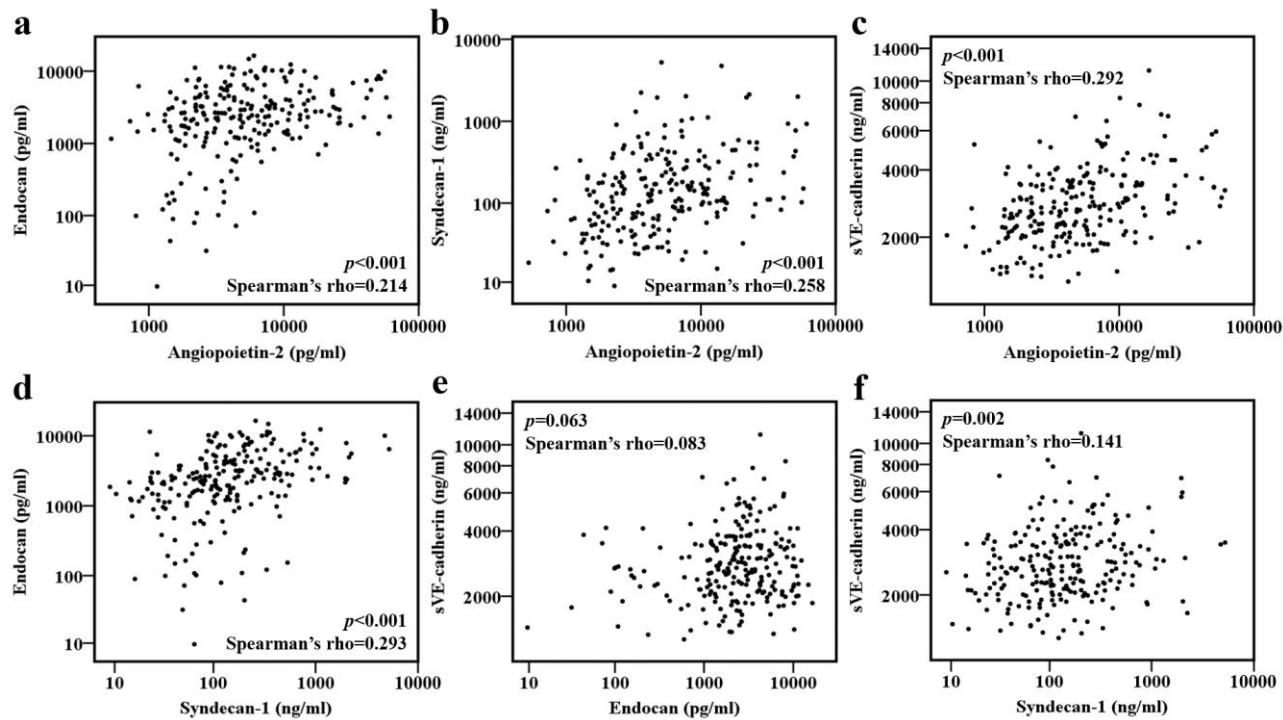

Supplement: Supplementary file 2 — Additional file 2. Plasma levels of angiopoietin-2 were modestly associated with (a) plasma levels of endocan, (b) plasma levels of syndecan-1 and (c) plasma levels of sVE-cadherin. Plasma levels of endocan were modestly associated with (d) plasma levels of syndecan-1, but not associated with (e) plasma levels of sVE-cadherin. Plasma levels of sVE-cadherin were modestly associated with (f) plasma levels of syndecan-1. Collinearity of these four endothelial biomarkers was analyzed by Spearman rank correlation test. [file 13054_2021_3474_MOESM2_ESM.pdf]

Supplementary figure 3

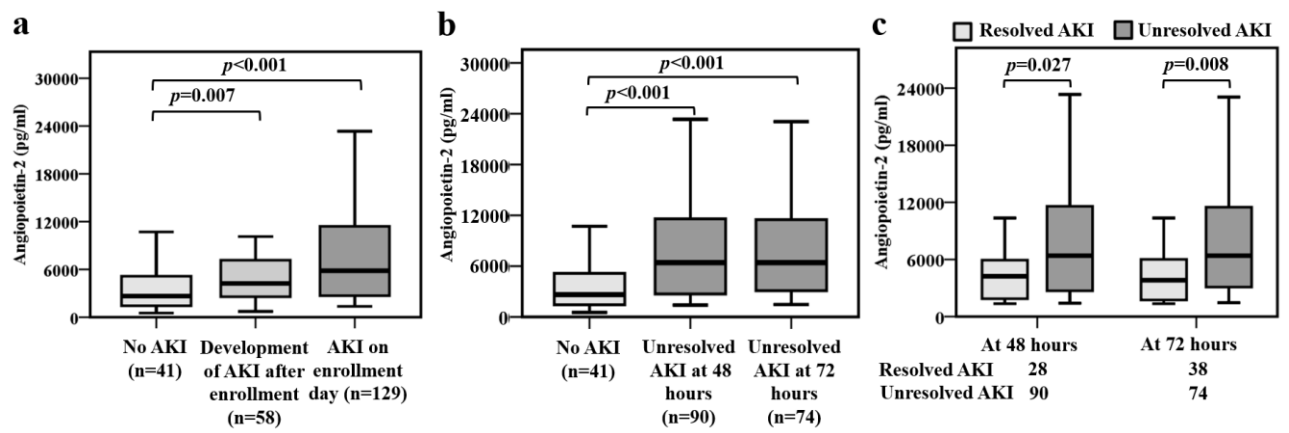

Supplement: Supplementary file 3 — Additional file 3. (a) Plasma levels of angiopoietin-2 were significantly lower in sepsis patients who did not develop AKI within the four study days compared to patients who had AKI on enrollment day or patients who developed AKI in the subsequent 72 hours after enrollment. (b) Plasma levels of angiopoietin-2 were significantly lower in sepsis patients without any AKI within the four study days compared to sepsis patients with persistent AKI at 48 or 72 hours since enrollment. (c) Among patients with AKI on enrolment day, plasma levels of angiopoietin-2 were significantly lower in patients with the resolution of AKI compared to patients without the resolution of AKI at 48 hours or 72 hours after enrollment. Data in panels a–c were summarized as boxplots where box encompassed 25‒75th percentile, error bars encompassed 10‒90th percentile and horizontal line showed median. Groups were compared by Kruskal-Wallis test (panels a and b) or Mann-Whitney U test (panel c). Post hoc analysis of groups comparison was performed using Mann-Whitney U test and Bonferroni correction (panels a and b). [file 13054_2021_3474_MOESM3_ESM.pdf]

Supplementary figure 4

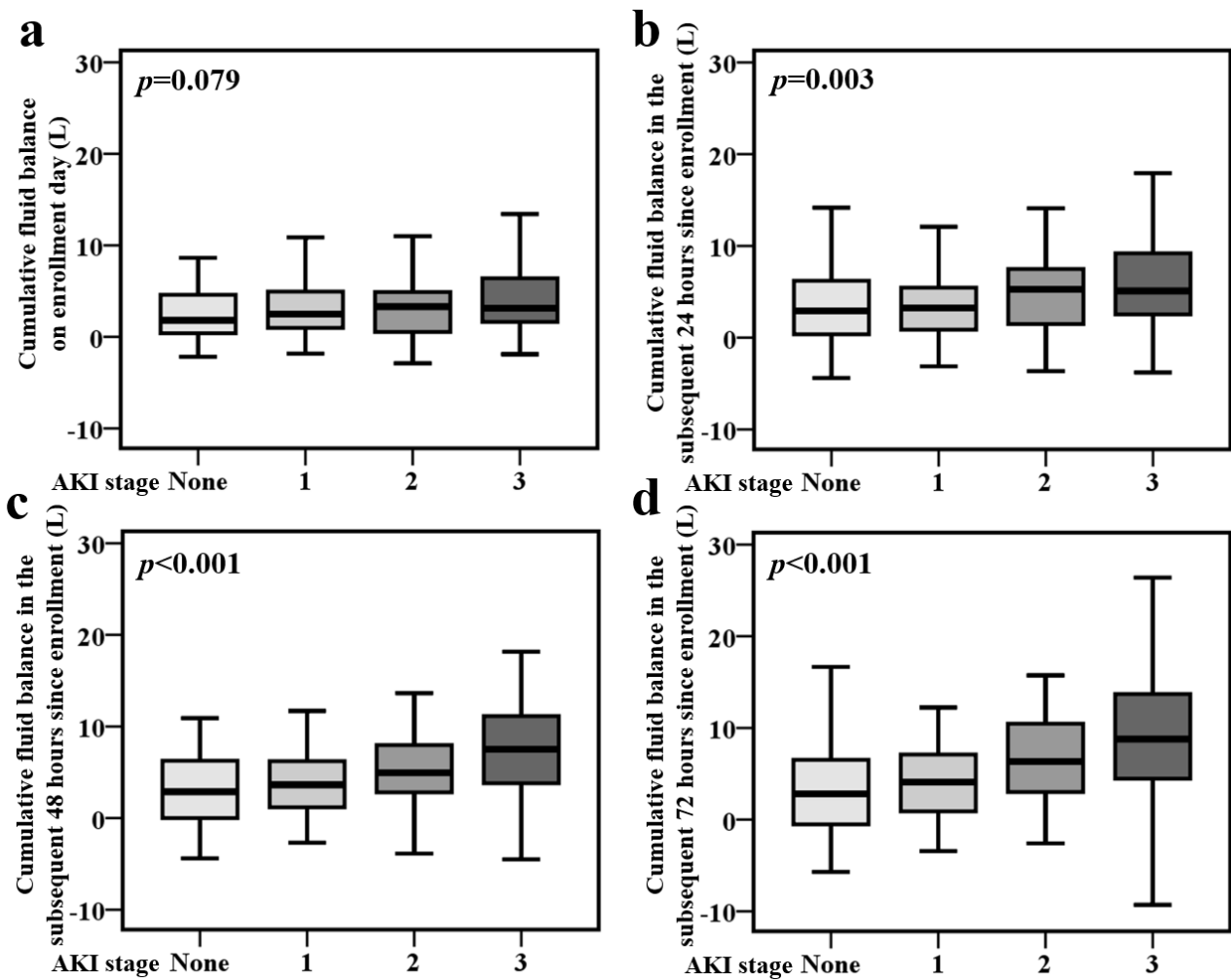

Supplement: Supplementary file 4 — Additional file 4. (a) The severity of AKI within the four study days was not associated with positive cumulative balance on enrollment day. The severity of AKI within the four study days was significantly associated with positive fluid balance in the subsequent (b) 24 hours, (c) 48 hours and (d) 72 hours since enrollment. Data in panels a-d were summarized as boxplots where box encompassed 25‒75th percentile, error bars encompassed 10‒90th percentile and horizontal line showed median. Groups were compared by Kruskal-Wallis test (panels a–d). [file 13054_2021_3474_MOESM4_ESM.pdf]

Supplementary figure 5

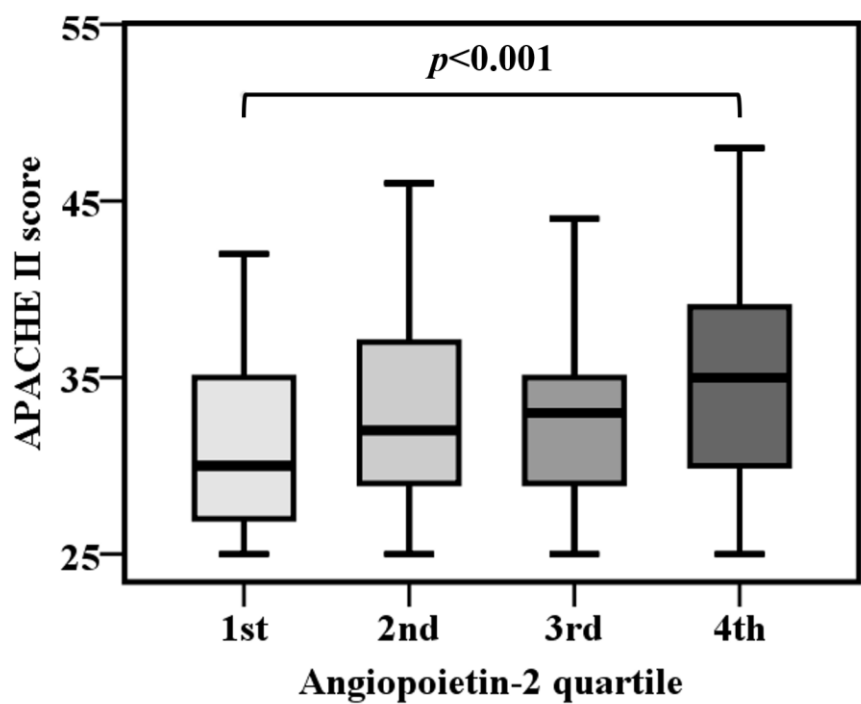

Supplement: Supplementary file 5 — Additional file 5. Higher plasma angiopoietin-2 levels by quartile were associated with higher APACHE II scores. Data were summarized as boxplots where box encompassed 25‒75th percentile, error bars encompassed 10‒90th percentile and horizontal line showed median. Groups were compared by Kruskal-Wallis test. Post hoc analysis of groups comparison was performed using Mann-Whitney U test and Bonferroni correction. [file 13054_2021_3474_MOESM5_ESM.pdf]
